# Acute pain intensity monitoring with the classification of multiple physiological parameters

**DOI:** 10.1007/s10877-018-0174-8

**Published:** 2018-06-26

**Authors:** Mingzhe Jiang, Riitta Mieronkoski, Elise Syrjälä, Arman Anzanpour, Virpi Terävä, Amir M. Rahmani, Sanna Salanterä, Riku Aantaa, Nora Hagelberg, Pasi Liljeberg

**Affiliations:** 1 Department of Future Technologies, University of Turku, Turku, Finland; 2 Department of Nursing Science, University of Turku, Turku, Finland; 3 Department of Computer Science, University of California Irvine, Irvine, USA; 4 Institute of Computer Technology, TU Wien, Vienna, Austria; 5 Turku University Hospital, Turku, Finland; 6 Pain Clinic, Turku University Hospital, Turku, Finland

**Keywords:** Acute pain intensity monitoring, Physiological parameters, Facial surface electromyogram, Artificial neural network

## Abstract

Current acute pain intensity assessment tools are mainly based on self-reporting by patients, which is impractical for non-communicative, sedated or critically ill patients. In previous studies, various physiological signals have been observed qualitatively as a potential pain intensity index. On the basis of that, this study aims at developing a continuous pain monitoring method with the classification of multiple physiological parameters. Heart rate (HR), breath rate (BR), galvanic skin response (GSR) and facial surface electromyogram were collected from 30 healthy volunteers under thermal and electrical pain stimuli. The collected samples were labelled as *no pain, mild pain* or *moderate*/*severe pain* based on a self-reported visual analogue scale. The patterns of these three classes were first observed from the distribution of the 13 processed physiological parameters. Then, artificial neural network classifiers were trained, validated and tested with the physiological parameters. The average classification accuracy was 70.6%. The same method was applied to the medians of each class in each test and accuracy was improved to 83.3%. With facial electromyogram, the adaptivity of this method to a new subject was improved as the recognition accuracy of *moderate*/*severe pain* in leave-one-subject-out cross-validation was promoted from 74.9 ± 21.0 to 76.3 ± 18.1%. Among healthy volunteers, GSR, HR and BR were better correlated to pain intensity variations than facial muscle activities. The classification of multiple accessible physiological parameters can potentially provide a way to differentiate among *no, mild* and *moderate*/*severe* acute experimental pain.

## Introduction

In acute pain management, the presence and intensity of pain are evaluated for decisions on intervention. Current pain intensity evaluation relies on patient self-reporting with tools like the visual analogue scale (VAS) and numerical rating scale. These one-dimensional assessment tools are considered powerful in acute pain assessment but require interactive communication between patient and caregiver, which is difficult for patients with limited communication capability [1]. Various physiological signals have been observed as potential pain intensity indicators, including heart rate (HR), heart rate variability, breath rate (BR), galvanic skin response (GSR) and photoplethysmographic pulse wave amplitude [2–10]. These parameters are easy to access in routine monitoring or with apparatus of a small size. However, there is unconformity in existing studies with different acute pain stimuli and subject groups in terms of the feasibility and superiority of each physiological parameter. Moreover, the variation of a sole physiological parameter is not specific to nociception, which is also influenced by other factors such as different physiological and psychological conditions, measurement and anesthesia [11]. A comprehensive index combined with multiple parameters analysis may lead to better pain intensity prediction than an individual parameter as more than one intensity category has the potential to be differentiated with comprehensive decision support information [12–15].

In addition to the above-mentioned physiological signs, the impact of facial expressions on pain intensity assessment is important. Facial expressions of pain can act as a behavioural source of evidence to mirror self-report ratings [16]. Ekman and Friesen [17] have decoded spontaneous facial expressions into the movements of individual facial muscles with a Facial Action Coding System (FACS). Through FACS, high accuracy in binary pain expression classification (pain versus no pain) can be reached [17, 18]. As to fine-sorted pain intensity levels, Prkachin and Solomon’s pain intensity (PSPI) scale is defined according to four facial actions (brow lowering, orbital tightening, levator contraction and eye closure) and is the sum of these four actions’ intensities [17–20]. With the help of computer vision algorithms and machine learning methods, automatic pain monitoring [21, 22] and continuous pain intensity estimation [22, 23] have been explored. However, from the perspective of practical use, the correspondence between the PSPI scale and VAS has not been clearly established, and from the perspective of reliability, the performance in existing studies is barely satisfactory.

To develop a continuous pain monitoring method from multiple physiological parameters with machine learning, HR, BR, GSR and facial surface electromyogram (sEMG) were monitored from healthy volunteers under experimental pain stimulus. Facial expressions were captured from sEMG of the skin above five pain expression-related facial muscles [24]: corrugator supercilii, orbicularis oculi, levator labii superiors, zygomaticus major and risorius. Two types of experimental pain stimuli, thermal stimuli (heat) and electrical stimuli, were employed on both the right and left sides of the body in the study to cover more than one dimension of pain perceptions [25]. Three pain intensity levels—*no pain, mild pain*, and *moderate*/*severe pain*—were collected from self-reports with VAS and were defined as three categories in classification.

## Methods

The study was approved by the Ethics Committee of the Hospital District of Southwest Finland (ETMK:83/1801/2015).

### Study protocol

#### Subjects and exclusion criteria

The study subjects were recruited by inviting generally healthy, voluntary working-age people. Each study subject provided a written informed consent. Thirty volunteers with no chronic or acute somatic or mental illness, taking no regular medication during the study or 2 weeks preceding it were included in the study. All the study subjects had normal cardiovascular parameter limits, normal sensation and healthy skin in the face and upper extremities. Pregnant subjects or subjects with a body mass index > 30 kg/m^2^ were excluded from the study.

#### Experimental pain stimuli

**Fig. 1:**
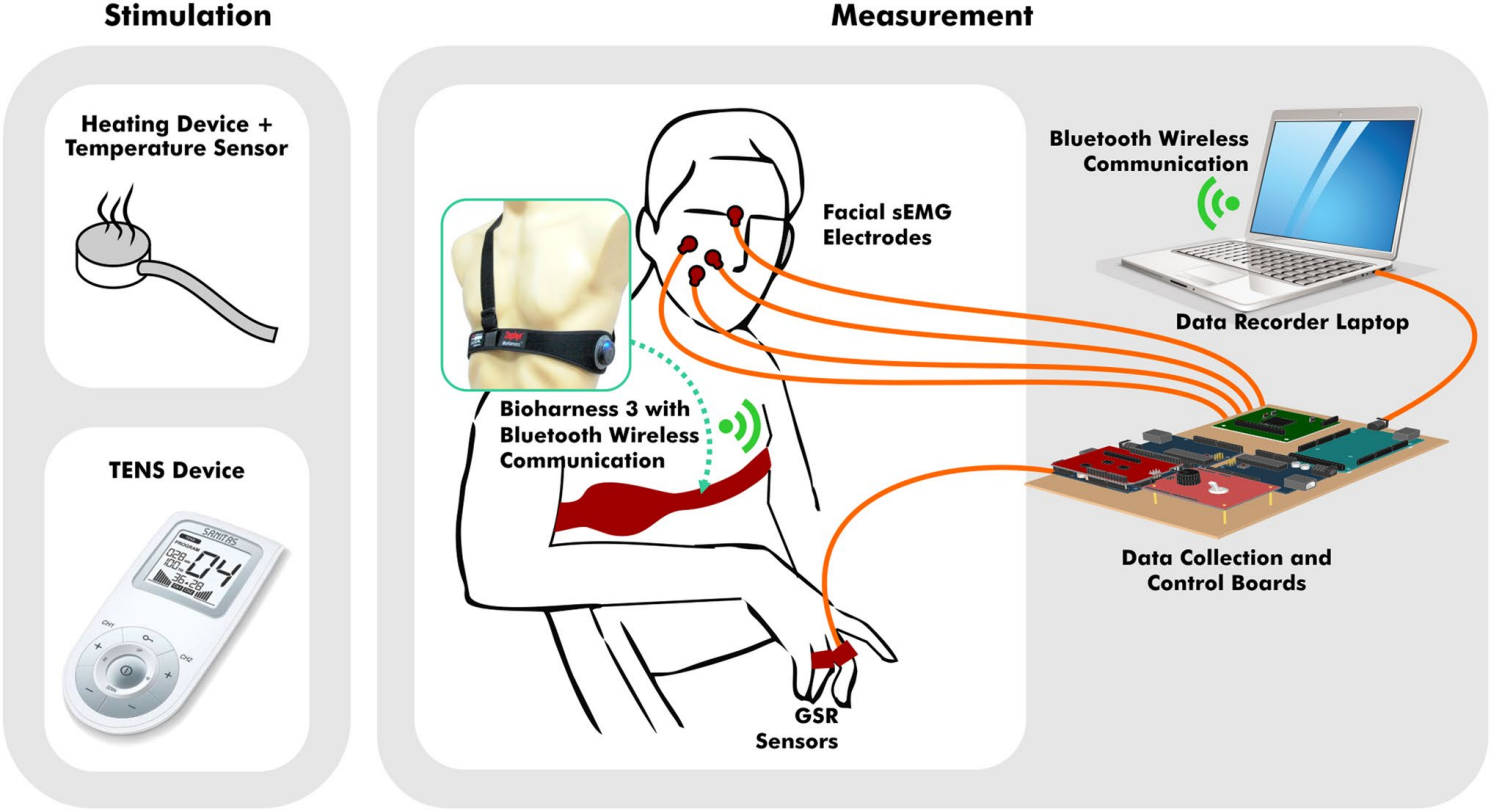
The devices for pain stimulation and the biopotential measurement environment

Slow heating (< 1℃/s) was chosen as a pain model. Heating the skin first activates the A-delta fibres and then the C-fibres, causing two types of pain. Slow heating mainly activates the C-fibres, causing longer pain stimuli which are less localized than with rapid heating [26]. The thermal pain was induced with a small heating element placed on the study subject’s inner forearm. The heating element was of a round shape with a diameter of 3 cm (shown in Fig. 1) and its temperature was controlled to increase 1 °C every 3 s up to 45 °C and every five seconds after that. The heating process stopped when reaching 52 °C to avoid skin burn and the temperature of the heating element then rose to 54 or 55 °C before cooling down. The heating element was detached from the skin surface of forearm immediately when the study subject reported an intolerable sensation. In the case that no intolerable sensation was reported during the temperature increment, the heating element was removed when reaching its maximum temperature. A cold pad was placed on the heated spot after each thermal pain test. The second pain stimulus was electrical stimulation [27]. This model was chosen because pain is only present during the stimulation. The electrical stimulus was induced on the fingertip of the ring finger with a transcutaneous electrical nerve stimulation (TENS). TENS is the use of an electrical neurostimulation in physical therapy and TENS device is available on the commercial market. The electrical stimulus is non-invasive and can be standardized. In the study, the electrical pulses from a Sanitas SEM 43- Digital EMS/TENS device were set at a width of 250 µs and repeated 100 times per second. TENS output has 50 intensity levels with a 200 milliampere maximum peak-to-peak current output when the load resistance is 500 ohms. The intensity of electrical pulses was controlled to increase level by level every three seconds until either the study subject could not tolerate the pain anymore or it reached the maximum level of 50.

#### Biopotential measurement

Physiological signals including HR, BR, GSR and five facial sEMG from the right side of the face were continuously recorded throughout the session. Figure 1 showed a brief description of the measurement environment, where GSR was captured from pre-gelled Ag/AgCl electrodes on the finger, five channels sEMG were captured from electrodes in the same type on corrugator supercilii, orbicularis oculi, levator labii superiors, zygomaticus major and risorius on the face, and HR and BR were from a Bioharness® belt worn on chest. HR, BR and GSR were taken at 1 s time resolution and sEMG were sampled with a Texas Instruments 8 channel biopotential measurement device at a rate of 1000 samples per second.

#### Study design

The study subject was seated in an armchair. At the beginning of the study session, the sensors and the device were established and it was ensured that they were able to record and appropriately catch the signals from all devices. The pain was induced by thermal and electrical stimuli in a random fashion, two times for each stimulus. The subjects were tested four times during each session and the tests were (1) electrical stimuli on the right-hand ring finger, (2) electrical stimuli on the left-hand ring finger, (3) thermal stimuli on the right inner forearm, and (4) thermal stimuli on the left inner forearm. The pain exposure starting location was randomized and the change of stimulated skin site helped in avoiding habituation to repeated experimental pain [28]. Each data collection session started by letting the subject settle down and rest for ten minutes, so as to acquaint himself or herself with the study environment. Pain testing was only repeated after the subject’s HR and BR had returned (if changed) to their respective baseline level.

**Fig. 2:**
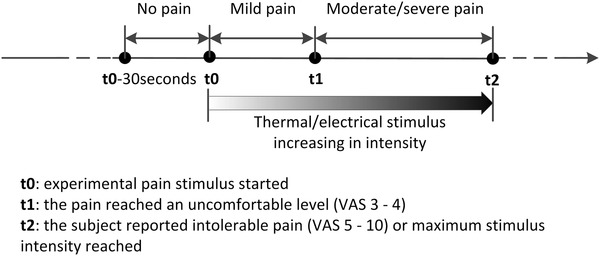
The timeline of one test, the time to have VAS report (t1 and t2) and the data label in each part (*no pain, mild pain* and *moderate*/*severe pain*)

The intensity of pain was evaluated using VAS at two time-points: t1—when the pain reached an uncomfortable level (VAS 3–4) and t2—when the study subject reported intolerable pain or when stimulus intensity reached the non-harmful maximum. The time points and data definition are illustrated in Fig. 2. To balance the data size of each class, data of the 30 s before applying pain stimulus was labelled as *no pain*. During pain stimulation, data from when it started to when it reached an uncomfortable level was labelled as *mild pain*. The second part of the data under pain stimulus was marked as *moderate*/*severe pain*, where either *moderate* or *severe* depends on the VAS the study subject reported. All physiological signals were marked with time stamps and were saved for offline processing along with VAS evaluations.

### Data pre-processing

**Fig. 3:**
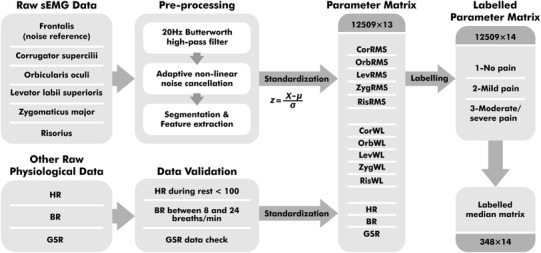
The data processing flow and two matrixes for classification

Data on sEMG and other physiological data were processed and checked separately, as shown in Fig. 3. The aim of the pre-processing was to eliminate noise interference and verify the validation of the data.

For sEMG, 50 Hz power line noise was coupled to electrode lead wires from the environment. Movement artifacts and baseline drift in low frequencies both caused noise in the sEMG signal. There was also a third noise source, which was caused by electrical stimulus pulses. Electrical pulses were added to finger skin’s surface and captured from facial skin’s surface as well, due to the electrical conductivity of the human body. In sEMG pre-processing, a 20 Hz Butterworth high-pass filter was first applied to remove movement artifacts and baseline drift from six sEMG channels. Adaptive noise cancellation was employed for the power line and electrical pulse elimination, where non-linear noise in each of the five pain-related facial muscle channels was estimated by reference to a frontalis sEMG with an adaptive neuro-fuzzy inference system (ANFIS) estimator [29].

To unify the time granularity of sEMG data and other physiological data, sEMG data was split into 1000-sample segments for feature extraction. The root mean square (RMS) in Eq. 1 and wavelength (WL) in Eq. 2 were the chosen features, where *N* was the window length and *x*_i_ was the *i*th data point in the window. The RMS feature provided direct insight on sEMG amplitude in order to provide a measure of signal power, while WL was related to both waveform amplitude and frequency [30]. All signal processing was conducted in MATLAB.

1 $$RMS=\sqrt {\frac{1}{N}\mathop \sum \limits_{{i=1}}^{N} x_{i}^{2}}$$

2 $$WL=\mathop \sum \limits_{{i=1}}^{{N - 1}} |{x_{i+1}} - {x_i}|{\text{~}}$$

For all physiological features, data validation on their range and conditions were carried out. After checking, three thermal stimuli tests were excluded from the total of 120 tests due to invalid GSR data in the *no pain* part and another thermal stimulus test was excluded for invalid sEMG data. All the validated physiological features were standardized with a standard core within each test and constituted the 13-dimensional parameter matrix. This standardization rescaled the range and distribution of each parameter, in which way the within-subject and the between-subject difference in value range were suppressed. There were 12,509 samples at one-second resolution from 116 tests in the parameter matrix. Each sample with 13 parameters was labelled according to the data division in Fig. 2. *No pain, mild pain* and *moderate*/*severe pain* data were labelled as 1, 2 and 3 respectively. Subsequently, the statistical median of every parameter was calculated from three sections of each test and constituted the median matrix with a length of 348.

### Data observation and classification

To visualize the median matrix in 2-dimensional scatter plots, the dimension of parameters in the median matrix was first reduced from 13 with principal component analysis. The first two principal components of the median matrix were non-normally distributed. Nevertheless, with the ability of multivariate analysis, Gaussian distributions were then estimated for each pain intensity level to observe their approximate distribution boundaries in the first two principal components. To fit Gaussians to the parameters of each group, the mean (*µ*) and variance (*σ*^2^) of Gaussian distribution were estimated in maximum likelihood estimation. In a *d*-dimensional Gaussian distribution, mean and variance were estimated from

$$\begin{gathered} {{\hat {\mu }}_i}=\frac{1}{N}\sum\nolimits_{{(n=1)}}^{N} {{x_{ni}},\quad {\text{for}} \quad \,i=1, \ldots d\,{\text{and}}} \hfill \\ {{\hat {\sigma }}^2}_{{ij}}=\frac{1}{N}\sum\nolimits_{{(n=1)}}^{N} {({x_{ni}} - {{\hat {\mu }}_i})({x_{nj}} - {{\hat {\mu }}_j}), \quad \,{\text{for}}\quad \,i,j=1, \ldots ,d.} \hfill \\ \end{gathered}$$

The 95% confidence regions of distributions were marked as approximate boundaries. Tests with different pain stimuli were plotted separately.

In the next observation, the significance of each parameter in pain intensity level recognition was observed with correlation analysis. Pearson’s linear correlation coefficients between each standardized parameter and labels were calculated.

Using the classification method in machine learning, a model can be built to predict class labels (i.e. 1—*no pain*, 2—*mild pain* and 3—*moderate*/*severe pain*) from input features (i.e. parameter matrix or median matrix). The resulting classifier is then used to assign class labels to the testing instance with new input features. One benefit of applying classification is its effectiveness in establishing the many-to-many mapping. The classification technique chosen in this study was the artificial neural network (ANN), which is a non-linear classifier having generally better performance with continuous and multi-dimensional features [31]. This method emulates the information processing capabilities of human brain neurons and can provide a flexible mapping between inputs and outputs [32].

**Fig. 4:**
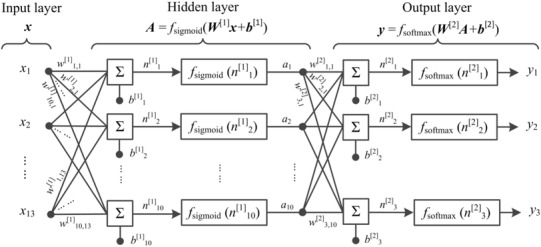
The ANNs classifier architecture including an input layer (***x*** = *x*_1,_*x*_2,…,_*x*_13_), a hidden layer (***A*** = *a*_1,_*a*_2,…,_*a*_10_) and an output layer (***y*** = *y*_1_, *y*_2_, *y*_3_)

With 13 parameters as the classifier inputs (***x***) and 3 pain intensity levels as the outputs (***y***), the ANNs classifier was built in three layers: an input layer with 13 units, a hidden layer with 10 units and an output layer with 3 units. As the architecture presented in Fig. 4, model parameter matrices ***W***^[1]^, ***b***^[1]^, ***W***^[2]^ and ***b***^[2]^ were optimized during training. The classifier was applied to both the labelled median matrix and the labelled parameter matrix. Before classification, the samples were divided randomly into three proportions, where 70% were training samples being presented initially to the classifier for training the network; 15% were validation samples to improve classifier generalization properly; and the remaining 15% were testing samples, independent from the trained classifier for classifier performance measurement. The classifier in this work was trained and evaluated in MATLAB Neural Network Toolbox® [33]. The receiver operating characteristic (ROC) curve of each classification was presented. Both average accuracy and the area under ROC curve (AUC) were evaluated as the performance of classification. The true positive rate (TPR) was also taken into consideration in the evaluation, indicating the correct recognition rate of each pain intensity level. The distributions of AUC in the classification with a different number of involved parameters were then presented.

Leave-one-subject-out cross-validation classification with an ANNs classifier was next applied to the labelled median matrix and parameter matrix. In this classification, each subject was left out in turn as test samples in order to examine the performance of the classifier trained by samples from the rest of the subjects. This approach was to look into the diversity among subjects and also to examine the adaptivity of this method to fresh subjects. In addition to the same results were presented with the classification without cross-validation above, the TPR of each class and the average accuracy for every subject were compared in a row. Furthermore, the impact of facial sEMG parameters in pain intensity level recognition was investigated by comparing classification performance without and with facial sEMG parameters.

## Results

Thirty volunteers (n = 30, 15 males and 15 females) in healthy condition were included in the study and examined between December 2015 and April 2016. The mean age was 33 in women (SD = 11.9) and 35 in men (SD = 8.0). Each person experienced four tests successively (with electrical pain stimulus on right/left ring finger and with thermal pain stimulus on right/left forearm). In total, 116 tests were included in the data analysis. The other 4 were excluded due to invalid measures. The average time length of each test involved in the analysis was 108 s (SD = 39 s). The average time interval between two successive tests was 289 s (SD = 192 s).

### Comparison between data from two pain stimuli

The mean VAS reported at the end of each test was 7.0 (SD = 1.3). In general, the electrical stimulus provided sharper nociception than the thermal stimulus, as a shorter transition time in mild pain and a higher final VAS on average were shown in Table 1. Besides, more electrical stimulus tests than thermal stimulus ones reached pain tolerance within non-harmful stimuli intensity range. Pain tolerance was reached in 58 out of the total 60 electrical tests and in 38 out of the total 56 thermal tests. The subjectivity of pain perception was reflected by the variance in maximum stimulus intensity of pain tolerance under similar VAS.

**Table 1:** Statistics of the test record of the amount, length, stimulus intensity and pain intensity

Item	Electrical pain stimulus	Thermal pain stimulus
Number of tests	60	56
Time length in seconds		
*Mild pain*	16 (SD = 10)	68 (SD = 16)
*Moderate*/*Severe pain*	37 (SD = 30)	36 (SD = 20)
Mean intensity of stimulus		
Uncomfortable (t1)	6^a^ (SD = 3^a^)	48.7 °C (SD = 2.9 °C)
Intolerable/maximum stimulus intensity reached (t2)	16^a^ (SD = 9^a^)	52.9 °C (SD = 2.2 °C)
Mean intensity of pain (VAS 0–10)		
Uncomfortable (t1)	3–4	3–4
Intolerable/maximum stimulus intensity reached (t2)	7.3 (SD = 1.2)	6.6 (SD = 1.3)

^a^TENS intensity level, 0–50. The output of TENS device was biphasic rectangular pulses with a width of 250 µs at a frequency of 100 Hz. The peak to peak output voltage was 100V in maximum (2V/ Intensity level)

**Fig. 5:**
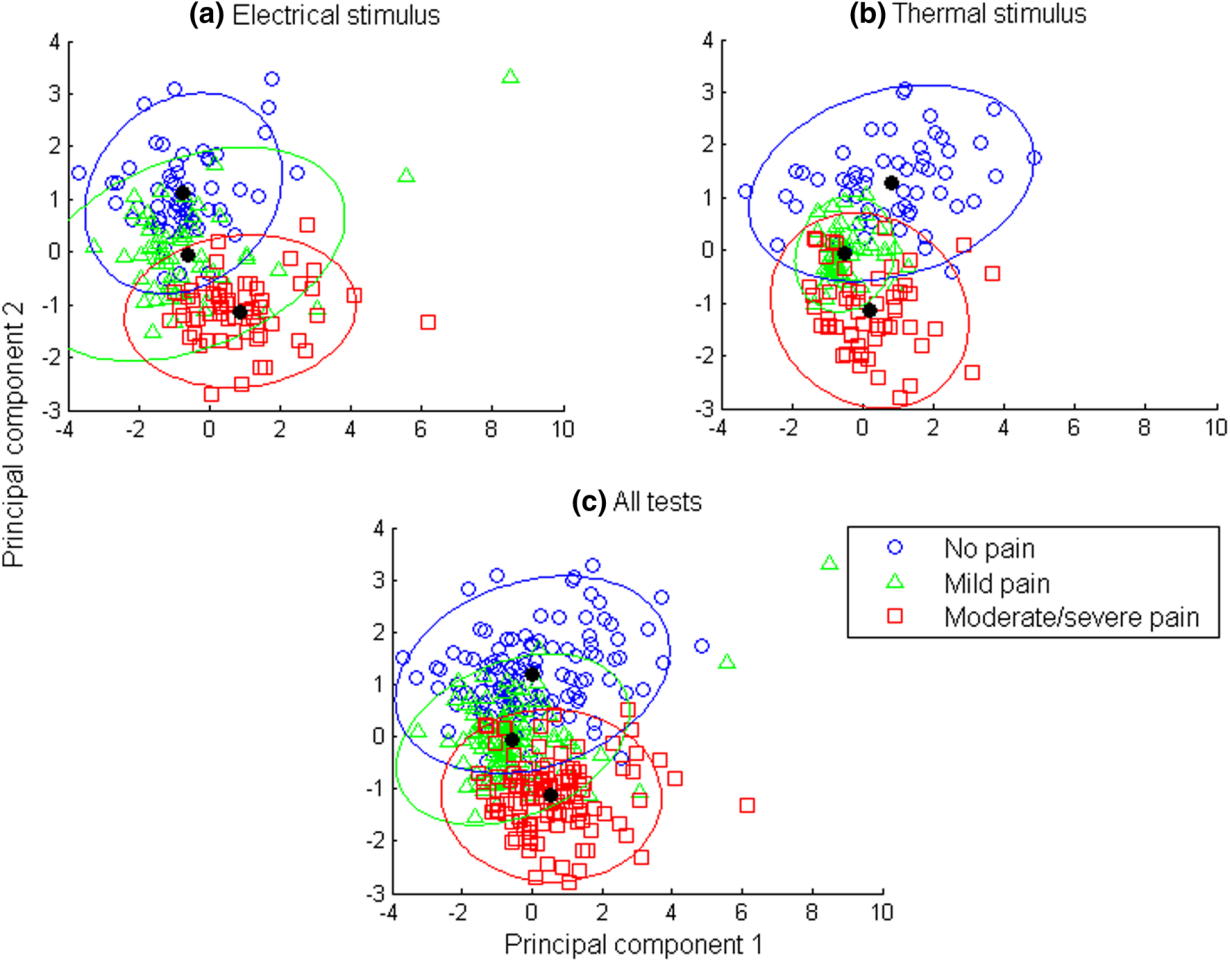
Median matrix is visualized in scatter plots after dimension reduction. Each data point stands for one sample in the median matrix and its shape represents its pain intensity level: the circles represent *no pain*, triangles represent *mild pain*, and squares represent *moderate*/*severe pain*. Tests with a single stimulus (Fig. 5a, b) and both stimuli (Fig. 5c) are visualized separately. The distribution of samples with the same pain intensity level is marked with its center and its 95% confidence region

To visualize the distribution of parameters, the 13-dimensional parameters are presented as scatter plot in a 2- dimensional space in Fig. 5. The median matrix was employed for data visualization and its dimension was reduced with principal component analysis. As shown in Fig. 5, samples in different pain intensity levels were presented with different markers. The Gaussian distribution of each sample group was estimated and marked with its center and 95% confidence region. Figure 5 shows that the centres of each group were clearly separated (one-way ANOVA between parameters and pain intensity level: *p* < 0.001) and approximately linearly distributed in pain intensity level, especially under an electrical stimulus (Fig. 5a). Data from the electrical stimulus (Fig. 5a) and thermal stimulus (Fig. 5b) followed similar relative relations among the three levels, where the samples labelled *no pain* separated relatively well with the samples labelled with *moderate*/*severe pain* while the samples labelled with *mild pain* distributed in between and overlapped with the other two groups. This was consistent with previous studies where pain tolerance could be highly differentiated from *no pain* with high binary classification accuracy, while lower accuracy was in the classification of multiple pain intensity among *no pain, pain threshold* and *pain tolerance* [34, 35].

Differences were observed between two pain stimuli. Samples of *moderate*/*severe pain* under electrical stimulus had less overlap with samples of *no pain* in Fig. 5a, compared to thermal stimulus in Fig. 5b. Meanwhile, *mild pain* samples under electrical stimulus was more dispersed (covariance matrix:$\left[3.2730 0.5505 0.5505 0.6838 \right]$, versus thermal stimulus $\left[0.3330 0.0314 0.0314 0.2022 \right]$). Although there were differences across pain stimuli, the median matrix from physiological parameters showed a pattern in pain intensity level, as shown in Fig. 5c.

### Correlation analysis

**Fig. 6:**
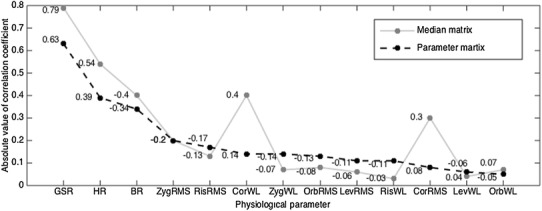
Pearson’s linear correlation coefficients between pain intensity levels (1—*no pain*, 2—*mild pain*, 3—*moderate*/*severe pain*) and parameters in the parameter matrix (the black dots) and between pain intensity levels and the parameters in the median matrix (the grey dots). The physiological parameters on the horizontal axis are sorted in descending order of coefficient absolute value. Corresponding signed coefficient values are marked

The Pearson’s linear correlation coefficients between parameters and pain intensity level are plotted in Fig. 6 in descending order of absolute value. Observation and comparison were made on both the parameter matrix and median matrix. GSR, HR and BR in the parameter matrix were presented to be the three best-predicting parameters among all the parameters in this study. GSR and HR were positively correlated with pain intensity level, indicating that these two parameters were more likely to increase when a healthy subject experiences a high intensity of pain, while BR decreases. Among facial sEMG parameters, ZygRMS had a higher correlation to the pain intensity level than others. As a measure of central tendency in parameters with less deviation, GSR, HR, BR and two corrugator superclii parameters in the median matrix showed stronger correlation to the pain intensity level than the parameter matrix. It was noticeable that medians of both corrugator supercilii parameters showed considerable potential for differentiating pain intensity levels. However, the same significance did not appear in the facial sEMG parameter matrix. This may suggest the transient response of facial expressions to acute pain.

### Classification with the median matrix and parameter matrix

**Fig. 7:**
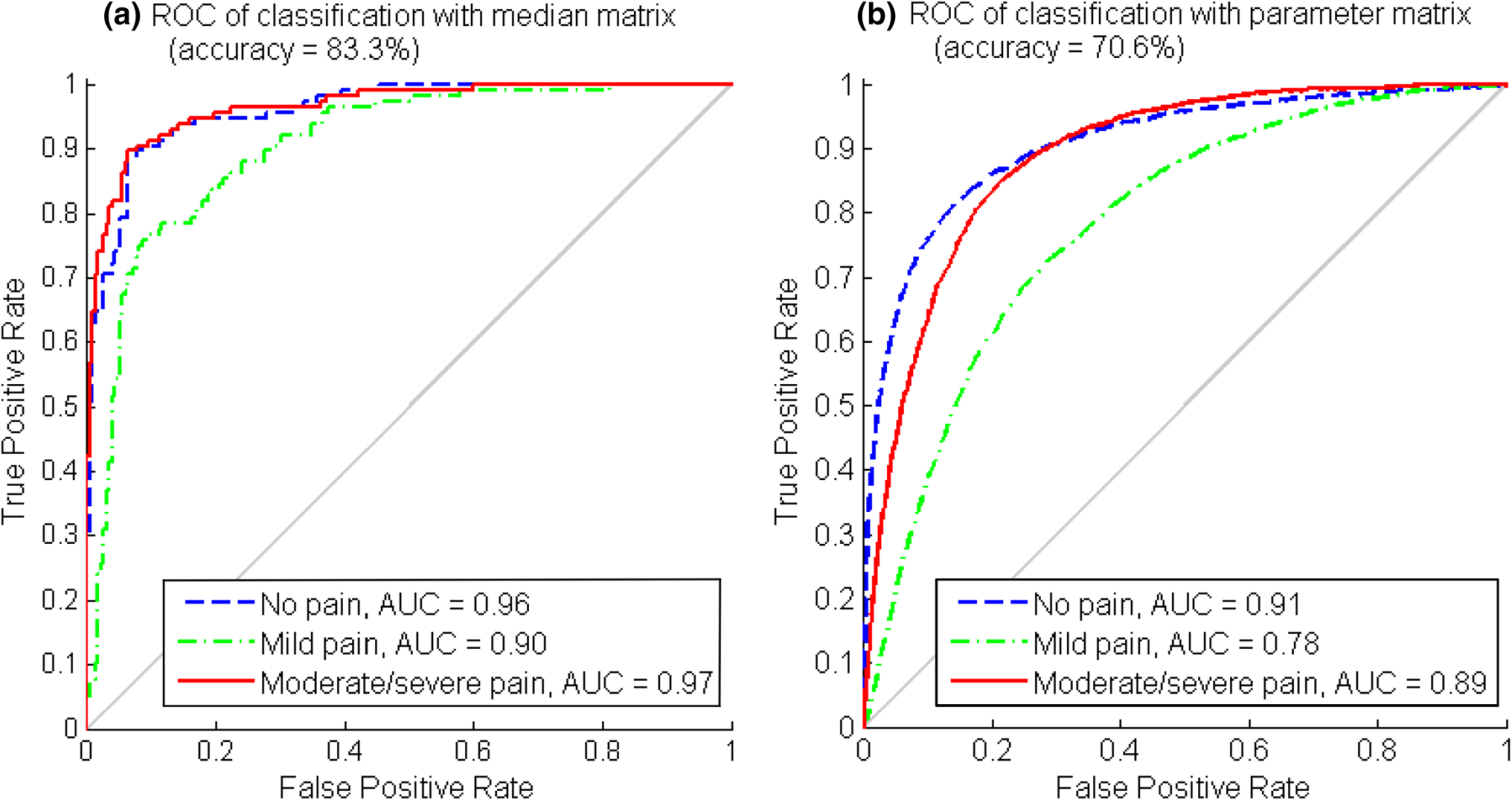
The ROC and AUC of each pain intensity level and the average accuracy in median matrix classification (Fig. 7a) and parameter matrix classification (Fig. 7b)

The ANNs classifier was trained, validated and tested with a median matrix and parameter matrix separately. The ROC curves from the overall classification results are presented in Fig. 7. The average classification accuracy of the median matrix reached 83.3%, where *no pain* TPR = 86.2%, *mild pain* TPR = 78.4%, and *moderate*/*severe pain* TPR = 85.3%. For parameter matrix classification, the average accuracy was 70.6%, where *no pain* TPR = 70.9%, *mild pain* TPR = 65.6%, and *moderate*/*severe pain* TPR = 70.6%.

Better classification results were obtained from median matrix classification than from parameter matrix classification. This was due to fewer fluctuations and less noise in the median matrix. However, classifying with the parameter matrix was closer to simulating pain intensity level monitoring in a real-time manner because no label was pre-known for the statistical median of each data section. Among the three pain intensity levels, the classifications of *no pain* and *moderate*/*severe pain* had the same good performance when compared to the *mild pain* classification. This corresponds to the data observation in Fig. 5, where *mild pain* samples were prone to be misclassified into the other two classes.

**Fig. 8:**
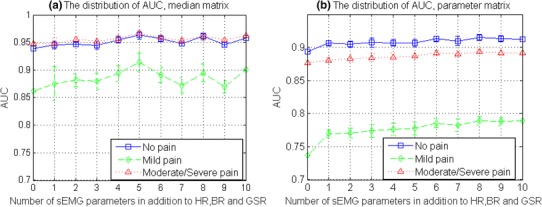
The distribution of AUC (mean ± SD) from classification with different number of sEMG parameters in addition to HR, BR and GSR, with all possible combinations. Results from the classification of median matrix are shown in Fig. 8a and results from the classification of parameter matrix are shown in Fig. 8b

According to the correlation analysis results, physiological parameters were the first three parameters that related most to pain intensity and were supposed to contribute the most to the classification performance. Therefore, the contribution of the sEMG parameters to the classification was next observed with all possible combinations. ANNs classifiers were trained and tested for each parameter combination. Their performance is presented in Fig. 8 with the distribution (mean ± SD) of AUC involving different numbers of sEMG parameters (from 0 to 10). It shows that adding sEMG features to classification in addition to physiological parameters improved the overall performance. With median matrix, the best performance appeared when 5 sEMG parameters were added to the classification. While with parameter matrix, AUC generally increased along with the increase of sEMG parameter amount, which was most prominent in *mild pain* (from 0.74 to 0.77 in average) when the number of sEMG parameters increased from 0 to 1.

### Leave-one-subject-out cross-validation

**Fig. 9:**
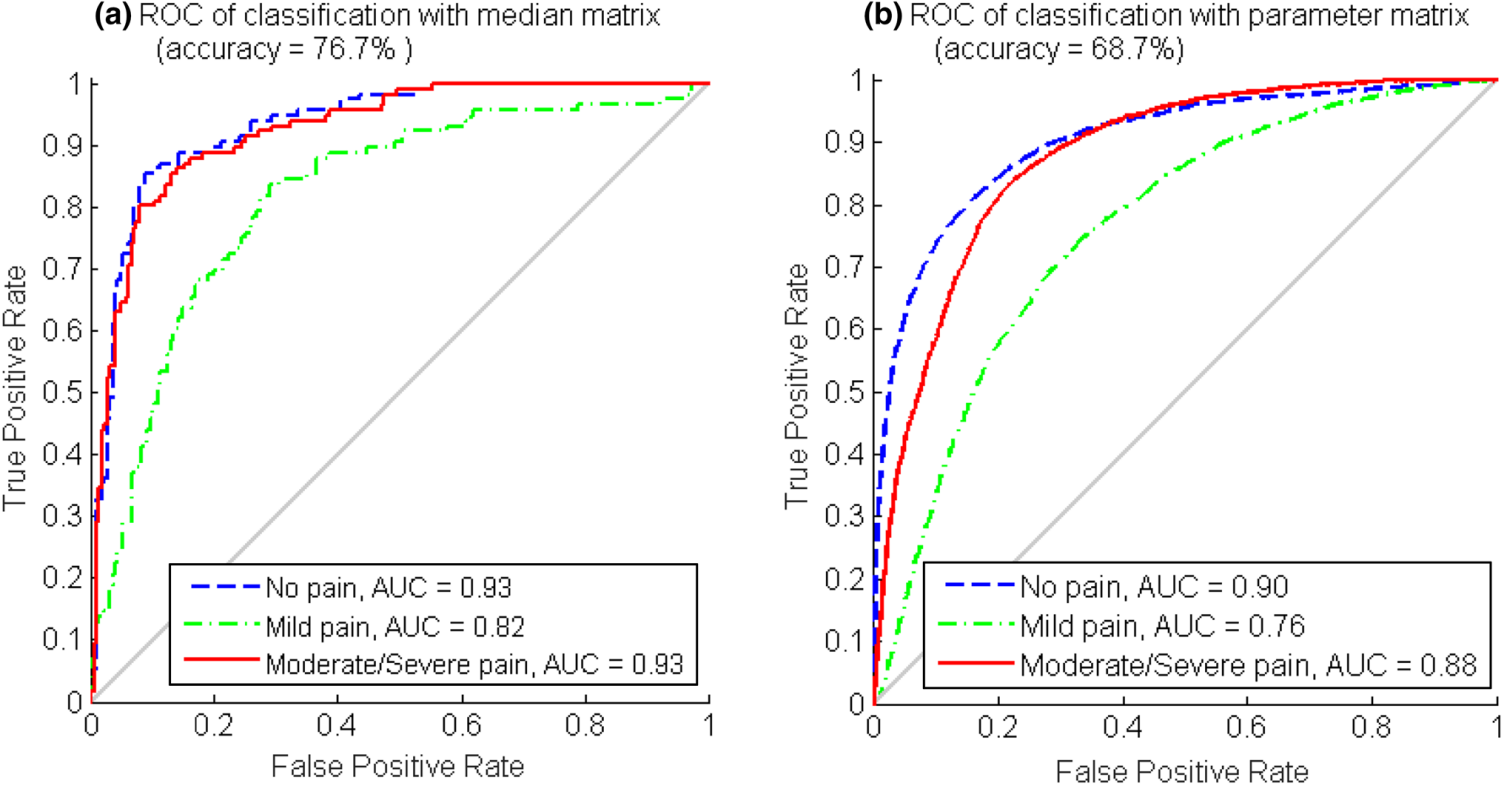
The ROC and AUC of each pain intensity level and the average accuracy with leave-one-subject-out cross-validation in median matrix classification (Fig. 9a) and parameter matrix classification (Fig. 9b)

The applicability of this pain intensity level recognition method to new subjects is examined in this part. A classification with leave-one-subject-out cross-validation was conducted among 30 subjects, where the neural network classifier was trained by the data from 29 subjects and its performance was examined by the data from the rest 1 subject. The results from the 13-dimensional parameter classification are shown in Fig. 9. Their performance yielded to the classification performance in Fig. 7, where data from one subject may be involved in both training data set and test data set. This may indicate the similarity of the data from the 4 within-subject tests and the dissimilarity among subjects.

**Fig. 10:**
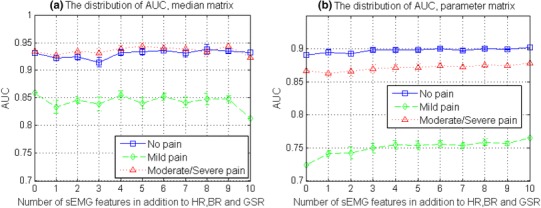
The distribution of AUC (mean ± SD) from leave-one-subject-out classification with a different number of sEMG parameters in addition to HR, BR and GSR, with all possible combinations. Results from the classification of the median matrix are shown in Fig. 10a and results from the classification of parameter matrix are shown in Fig. 10b

The contribution of sEMG parameters was then observed in the classification of both median matrix and parameter matrix with leave-one-subject-out cross-validation. The AUC distributions in Fig. 10 showed that sEMG parameters in parameter matrix helped improve the performance, while sEMG parameters in median matrix did not.

Furthermore, leave-one-subject-out classifications with facial sEMG parameters exclusively and inclusively were both conducted on the parameter matrix. The statistics of average accuracy and TPR from all subjects were placed in Table 2. It showed that on the basis of HR, BR and GSR, facial sEMG parameters contributed with both a mean accuracy improvement and individual deviation reduction. The mean TPR and accuracy across 30 subjects were slightly inferior to the parameter matrix classification results in Sect. 3.3, except for the *moderate*/*severe pain* level class where 76.3% of mean TPR was in the leave-one-subject-out classification and 70.6% in the overall classification. Classification performance varied among different subjects, reflected by the standard deviation of 10.5% in all classes and 18.1% in the *moderate*/*severe pain* level.

**Table 2:** TPR and accuracy in parameter matrix classification without and with facial sEMG parameters

Classification	No pain mean ± SD (%)	Mild pain mean ± SD (%)	Moderate/severe pain mean ± SD (%)	Accuracy mean ± SD (%)
HR + BR + GSR	68.1 ± 18.2	57.8 ± 10.0	74.9 ± 21.0	65.4 ± 13
HR + BR + GSR + sEMG	69.7↑ ± 16.0↓	64.6↑ ± 9.1↓	76.3↑ ± 18.1↓	68.2↑ ± 10.5↓

**Fig. 11:**
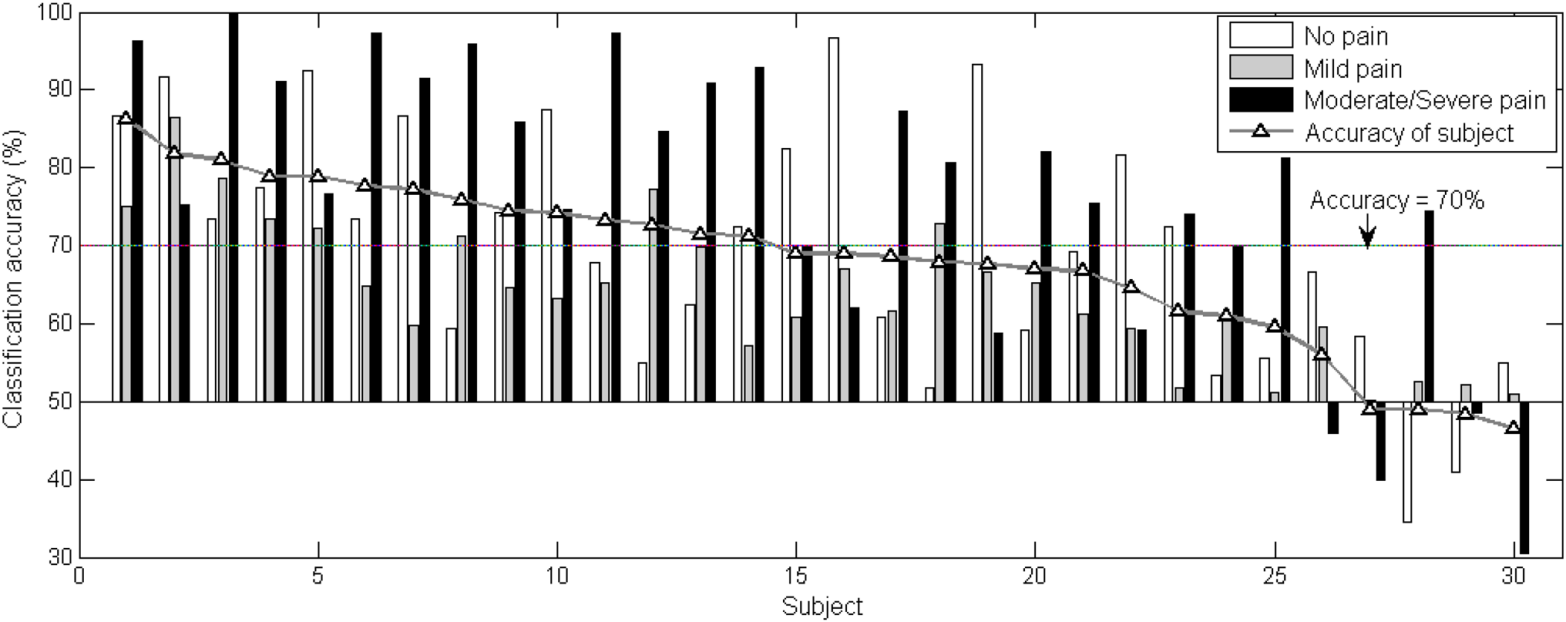
The TPR of each pain intensity level and average accuracy for each subject in leave-one-subject-out classification with a base value of 50%. Seventy per cent is taken as a characteristic value for performance evaluation

To observe the individual difference among subjects, the details of leave-one-subject-out classification with a 13-dimensional parameter matrix were plotted. The average accuracy of each test subject and the TPR of each pain intensity level is presented in Fig. 11, where the test subject was sorted in descending average classification accuracy It can be observed that most of the subjects had a pain intensity level predictable with average classification accuracy above 50%. With the multi-parameter classification method, half of the subjects had good classification performance with accuracy higher than 70%. Among the three classes, the *moderate*/*severe pain* was best predicted and was correctly recognized with a rate no less than 70% among 23 of all 30 subjects. The subjects with low classification accuracy may have shown a different reaction pattern to pain stimulation comparing to others.

### Results summary

In this work, patterns of self-reported acute pain intensity levels from monitored physiological signals were observed, which were categorized into *no pain, mild pain* and *moderate*/*severe pain* based on reported VAS. On the basis of that, a quantitative relation was mapped between physiological signals and pain intensity levels through classification. A classical supervised learning method was applied to the processed 13-dimensional physiological parameters under two experimental pain stimuli.

The distribution change, along with experimental acute pain stimulus intensity change, was first observed from a comprehensive group of monitored biopotentials in Fig. 5c. Differences in median matrix distribution were seen between tests under different stimuli in Fig. 5a, b. Among the 13 physiological parameters, HR, BR and GSR played an important role, while the role of facial sEMG parameters was comparatively less significant, according to the correlation analysis presented in Fig. 6. Nevertheless, the sEMG parameters still contributed to the parameter matrix classification as the number of them increased (Figs. 8, 10).

Secondly, as the median matrix showed generally higher relevance to pain intensity level, the processed parameters led to better pain intensity pattern and classification results, as shown in Fig. 7. The average classification accuracy of 83.3% with the median matrix was prior to study [35], with comparative lighter computation, and was close to the non-linear regression performance in [15] between multiple physiological parameters and an index combining both stimulus level and analgesic effect. In this study, the TPR of *moderate*/*severe pain* was of higher than average accuracy in both median matrix and parameter matrix classifications. Although there are data size limits, it is worth digging further into physiological parameter patterns with subdivided VAS in *moderate*/*severe pain*.

Thirdly, individual difference appeared as the classification accuracy difference in leave-one-subject-out cross-validation, as shown in Fig. 11. In addition, the overall performance of the classification with leave-one-subject-out cross-validation was inferior to the classification without cross-validation, comparing Fig. 9 to Fig. 7. Similarly, the performance difference between classification without cross-validation and classification with leave-one-subject-out cross-validation was also observed in [34, 36].

The classification of the parameter matrix was to imitate real-time continuous pain intensity monitoring. The basic competence of pain intensity prediction has been shown in this study. On the basis of these results, there is room for performance improvement in several aspects. One is using more dimensions of information in signal processing other than a simple statistical median. For example, the number of fluctuations, features of stationarity, entropy, similarity [15, 35, 37] and features in the frequency domain can be further applied. In the continuous monitoring manner, a time window with a fixed length can be added to streaming data. Data within the time window is processed and features are extracted for classification in the next step. Another aspect is the optimization of learning algorithm where classifier parameter optimization and feature selection can be applied. The use of deep learning may also contribute to performance improvement. A third aspect is the post-processing of classification results. For example, when looking into the results in a time span, some misclassification behaves such as swings between two classes and the results in this time period can be polished in order to be steady and accurate.

## Discussion

The results show the possibility of automatic pain monitoring by the classification of multiple physiological parameters. Furthermore, the results of parameter matrix classification show the potential of continuous pain monitoring, where physiological parameter samples were classified in every second. These physiological parameters are either clinically accessible or available from wearable devices [38, 39] and are appropriate for continuous and long-term monitoring. Besides, this monitoring method may help clinicians and nurses read patients’ acute pain and hence treat it more efficiently, especially for the patients unable to communicate verbally.

The exploring of automatic pain assessment markers was conducted by observing the autonomic nervous system activities [8, 9, 40] and brain responses [11]. It was indicated that a combination of several parameters with regression is more promising than any single one [14] and which was further validated in a clinical setting [15]. Additionally, the BioVid heat pain database [41] was established to analyze people’s reactions to experimental heat pain stimulus from a psychobiology point of view. Their following studies focused on feature extraction in data pre-processing and the fusion of facial expression video images with physiological parameters [34, 35, 42, 43]. This study proceeded to the continuous assessment of experimental pain from the continuous changing of physiological parameters.

Like most of the methods developed from experimental pain tests, there are challenges when this method is applied in clinical settings. One challenge can be the different truth-value of pain intensities or stimulus intensities defined across studies. In experimental pain studies, self-report is available with either VAS scale or pain threshold and pain tolerance due to the individual differences in the subjective experience of pain [44]. Self-report is inaccessible in some clinical settings where anesthesia is applied, and therefore either nociception levels are defined and used instead as the truth-value [15], or the objective of the monitoring is altered into nociception-antinociception balance instead [45]. Another challenge is the potential psychological state difference when experience experimental pain and clinical pain, where psychological factors have an important influence on pain perception [46, 47] and modulate physiological signals differently. Other challenges of applying this method to clinical monitoring may include the regulation or unavailability of physiological parameters as a result of medications and disease symptoms. This, however, can also be an advantage of using multiple parameters where the unregulated and available ones may compensate the absence of the others, which needs further exploration.

One limitation of the study was the contamination of electrical stimuli pulses of facial sEMG signals. Adaptive noise elimination can remove a considerable part of the noise. However, the muscle activity from frontalis could be considered as noise and be eliminated from the other facial sEMG channels as well. Another limitation of this study was the choice of *no pain* data, where 30 s before applying experimental the pain stimulus was considered but data after removing the pain stimulus was not taken into account. In addition, there was no stimulation in the *no pain* control condition, which would be more appropriate if had applied a non-painful stimulus.

To conclude, the classification of multiple physiological parameters can be a solution to automatic pain intensity assessment. The method is potential to provide monitoring in a continuous manner (e.g. with a time resolution of one second). In the continuous monitoring, a combination of parameters is superior to a single parameter in terms of classification performance, especially in *mild pain* whose data overlapped greatly with the other two categories. Nevertheless, this method should be further improved in its adaptivity to a fresh subject, as the classification performance varies in subject level in leave-one-subject-out cross-validation.

## Appendix

The classification results from the models built with one test per subject (“Test 1”/“Test 2”/“Test 3”/“Test 4”) are compared to the results from the model built with all tests per subject (“All tests”). “All tests” represents the model built with all the four tests from each subject. “Test 1” represents the model built with the first test from each subject. “Test 2”, “Test 3” and “Test 4” are similarly defined. The results of “All tests” in each table are copied from the main body of the paper. The result difference between “Test 1”/“Test 2”/“Test 3”/“Test 4” and “All tests” is calculated and presented in the brackets (Tables 3, 4, 5, 6).

**Table 3:** Median matrix classification without cross-validation, compared to Fig. 7a

	All tests	Test 1	Test 2	Test 3	Test 4
Data size	348	87	87	84	90
Average accuracy (%)	83.3	46.0 (− 37.3)	87.0 (+ 3.7)	84.5 (+ 1.2)	72.2 (− 11.1)
TPR of *no pain* (%)	86.2	44.8 (− 41,4)	93.1 (+ 6.9)	82.1 (− 4.1)	70.0 (− 16.2)
TPR of *mild pain* (%)	78.4	93.1 (+ 14,7)	82.8 (+ 4.4)	78.6 (+ 0.2)	66.7 (− 11.7)
TPF of *moderate*/*severe pain* (%)	85.3	0.00 (− 85.3)	86.2 (+ 0.9)	92.9 (+ 7.6)	80.0 (− 5.3)
AUC of *no pain*	0.96	0.91 (− 0.05)	0.99 (+ 0.03)	0.95 (− 0.01)	0.94 (− 0.02)
AUC of *mild pain*	0.90	0.51 (− 0.39)	0.94 (+ 0.04)	0.94 (+ 0.04)	0.78 (− 0.12)
AUC of *moderate*/*severe pain*	0.97	0.20 (− 0.77)	0.97 (0.00)	0.97 (0.00)	0.91 (− 0.06)

**Table 4:** Parameter matrix classification without cross-validation, compared to Fig. 7b

	All tests	Test 1	Test 2	Test 3	Test 4
Data size	12,509	2801	2972	3398	3338
Average accuracy (%)	70.6	72.4 (+ 1.8)	70.6 (0.0)	78.1 (+ 7.5)	72.4 (+ 1.8)
TPR of *no pain* (%)	70.9	80.1 (+ 9.2)	75.1 (+ 4.2)	74.9 (+ 4.0)	72.1 (+ 1.2)
TPR of *mild pain* (%)	65.6	53.9 (− 11.7)	68.9 (+ 3.3)	76.2 (+ 10.6)	73.6 (+ 12.0)
TPF of *moderate*/*severe pain* (%)	70.6	82.2 (+ 11.6)	68.4 (− 2.2)	82.6 (+ 10.0)	73.2 (+ 2.6)
AUC of *no pain*	0.91	0.93 (+ 0.02)	0.94 (+ 0.03)	0.94 (+ 0.03)	0.91 (0.00)
AUC of *mild pain*	0.78	0.78 (0.00)	0.79 (+ 0.01)	0.86 (− 0.08)	0.82 (+ 0.04)
AUC of *moderate*/*Severe pain*	0.89	0.91 (+ 0.02)	0.89 (0.00)	0.94 (+ 0.05)	0.89 (0.00)

**Table 5:** Median matrix classification with leave-one-subject-out cross-validation, compared to Fig. 9a

	All tests	Test 1	Test 2	Test 3	Test 4
Data size	384	87	87	84	90
Average accuracy (%)	76.7	70.1 (− 6.6)	67.8 (− 8.9)	77.4 (+ 0.07)	76.7 (0.0)
AUC of *no pain*	0.93	0.88 (− 0.05)	0.91 (− 0.02)	0.88 (− 0.05)	0.90 (− 0.03)
AUC of *mild pain*	0.82	0.72 (− 0.10)	0.71 (− 0.11)	0.87 (+ 0.05)	0.82 (0.00)
AUC of *moderate*/*severe pain*	0.93	0.88 (− 0.05)	0.85 (− 0.08)	0.94 (+ 0.01)	0.91 (− 0.02)

**Table 6:** Parameter matrix classification with leave-one-subject-out cross-validation, compared to Fig. 9b

	All tests	Test 1	Test 2	Test 3	Test 4
Data size	12,509	2801	2972	3398	3338
Average accuracy (%)	68.7	63.4 (− 5.3)	63.1 (− 5.6)	72.2 (+ 3.5)	66.8 (− 1.9)
AUC of *no pain*	0.9	0.89 (− 0.01)	0.71 (− 0.19)	0.81 (− 0.09)	0.86 (− 0.04)
AUC of *mild pain*	0.76	0.67 (− 0.09)	0.59 (− 0.17)	0.79 (+ 0.03)	0.75 (− 0.01)
AUC of *moderate*/*severe pain*	0.88	0.86 (− 0.02)	0.60 (− 0.28)	0.90 (+ 0.02)	0.84 (− 0.04)
